# Acral Melanosis with Macromelanosomes (AMM): The First AI-Defined Pathological Entity and Proposal of Provisional Diagnostic Criteria

**DOI:** 10.7759/cureus.97276

**Published:** 2025-11-19

**Authors:** Igor Shendrik, Svetlana Bobkova

**Affiliations:** 1 Dermatopathology Section, Pathology Laboratory Associates, Tulsa, USA; 2 School of Biomedical Sciences, Oklahoma State University Center for Health Sciences, Tulsa, USA

**Keywords:** acral skin, artificial intelligence, chatgpt, fontana‑masson, macromelanosomes, melan-a, melanosis, sox10

## Abstract

Artificial intelligence (AI) has started to transform diagnostic workflows in pathology, yet no formal pathological entity has been named, defined, and documented in the medical literature by an AI system. We report a benign acral pigmentary process characterized by conspicuous keratinocyte macromelanosomes with otherwise normal melanocyte density and architecture. We propose the term “acral melanosis with macromelanosomes (AMM),” a name and construct first generated with the assistance of a large language model (ChatGPT), and we present clinical, histologic, and special‑stain findings along with provisional diagnostic criteria.

## Introduction

Acral skin, the specialized, glabrous skin covering the palms, soles, and subungual areas, is characterized by a markedly thickened stratum corneum and a pronounced pattern of interdigitating rete ridges. In healthy acral skin, melanocytes are aligned along these ridges, preferentially located at their tips and sides; thus, melanocyte density appears higher than in non‑acral skin, even under normal conditions. Acral melanocytic lesions, whether benign or malignant, often show histologic features rarely observed in other areas of the skin. Acral lentiginous melanoma (ALM), in particular, may mimic benign lesions both clinically and histologically, posing significant challenges to clinicians and pathologists due to the subtle nature of early melanocytic proliferation confined to the dermoepidermal junction. Consequently, clinical suspicion is heightened when a new pigmented lesion arises on the sole or palm of an elderly individual, leading to biopsy even when the lesion appears morphologically banal. The clinical appearance is not distinctive; the value lies in the histologic pattern and its correlation.

The cytologic appearance of melanocytes in early atypical acral melanocytic lesions is often the critical clue for the early detection of evolving neoplasms, underscoring the importance of closely examining melanocytic morphology in pigmented acral lesions. One rarely reported feature is the presence of macromelanosomes, abnormally large melanin granules observed within melanocytes or keratinocytes. These granules may be many times the size of normal stage IV melanosomes and can exhibit irregular shapes or vacuolated borders. They are most readily recognized in congenital pigmentary disorders such as Chediak‑Higashi syndrome, Griscelli syndrome, and certain forms of albinism. Within the cutaneous pathology literature, giant melanin granules have occasionally been reported in ephelides (freckles), lentigo simplex, and café‑au‑lait macules; however, macromelanosomes are rarely described in acquired lesions. The presence of macromelanosomes in a new acral lesion of an elderly patient is particularly concerning, since both dysplastic nevi and ALM may demonstrate large melanin granules at their peripheries.

In recent years, digital and computational pathology has expanded rapidly, progressing from simple slide digitization to robust applications in triage, tumor detection, grading, and prognostication. Nonetheless, contemporary reviews emphasize their role in clinical assistance and biomarker discovery rather than in the establishment of entirely new nosologic entities.

This case report describes an acral pigmentary lesion in a 77‑year‑old male, in which the dominant histologic feature was keratinocyte macromelanosomes with otherwise preserved melanocyte number and architecture. Leveraging an artificial intelligence (AI) conversational system (ChatGPT‑5, OpenAI, San Francisco, CA), we formulated and refined the term “acral melanosis with macromelanosomes (AMM)” to capture this pattern and encourage prospective validation. Here, we present the clinicopathologic findings, summarize the relevant literature on macromelanosomes and acral pigmentation, and propose provisional diagnostic criteria and nomenclature [1-3].

## Case presentation

**Figure 1:**
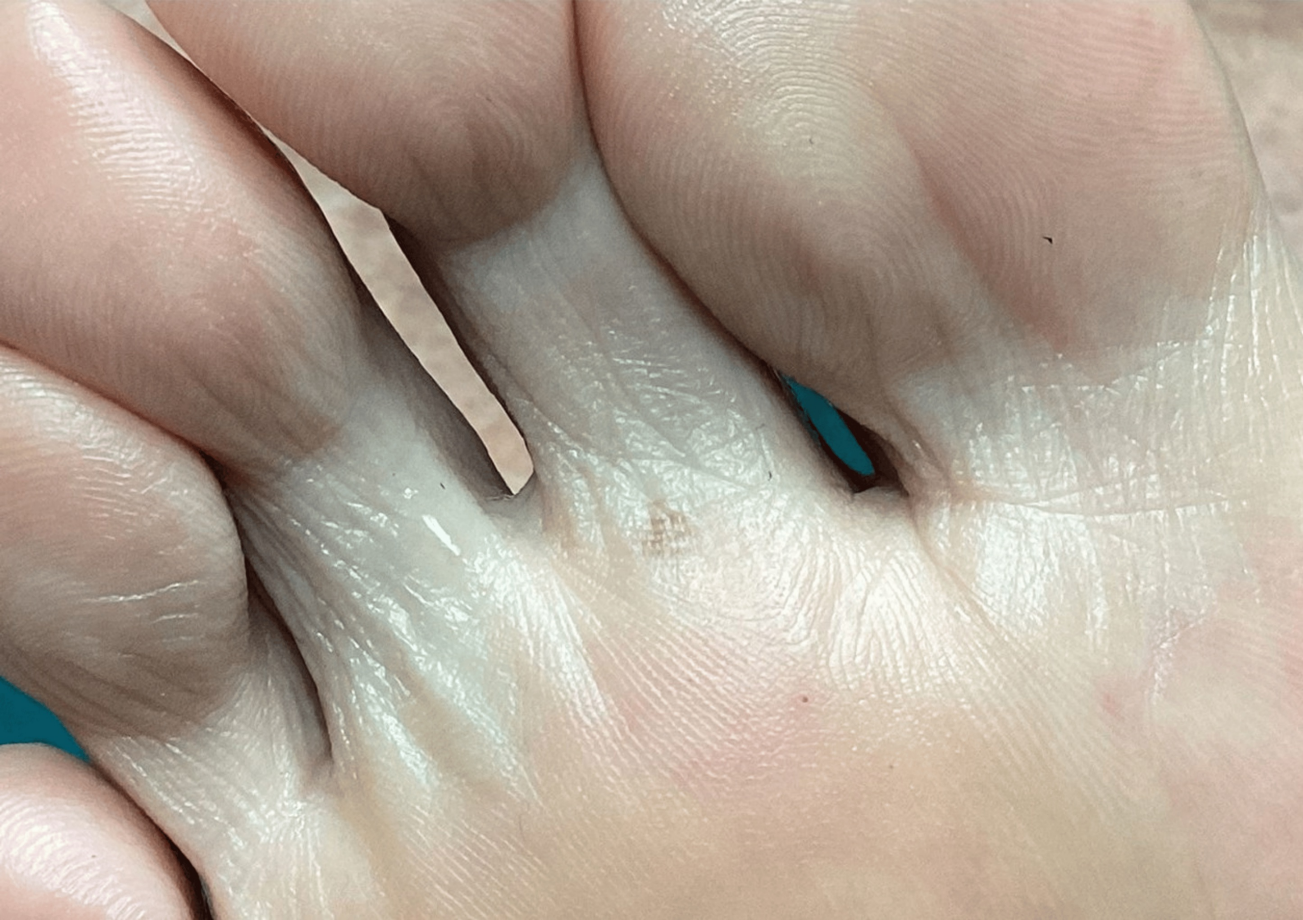
Clinical photograph of the right plantar surface demonstrating a well-demarcated 0.8 cm dark brown macule beneath the second metatarsophalangeal joint. The lesion shows uniform pigmentation without ulceration, bleeding, or secondary changes.

A 77-year-old non-Hispanic White man, Fitzpatrick skin type II, with a medical history notable for diabetic foot, presented for podiatric evaluation. He denied any complaints and reported no personal or family history of melanoma, dysplastic nevi, or pigmentary syndromes. On examination, an incidental, slowly evolving, asymptomatic patch of variegated brown pigmentation was noted on the plantar surface (Figure 1). The lesion had remained clinically stable for several months, without rapid enlargement, bleeding, or pain. On physical examination, an irregularly pigmented macule was observed on a weight‑bearing area of the forefoot, with indistinct borders. Dermoscopy did not reveal a parallel ridge pattern; instead, pigmentation appeared mottled, lacking linear ridging. The patient reported no history of chronic frictional activity at the site and no prior use of medications known to cause cutaneous pigmentation (e.g., minocycline, hydroxyurea, antimalarials). A shave biopsy was performed for histopathologic evaluation.

**Figure 2:**
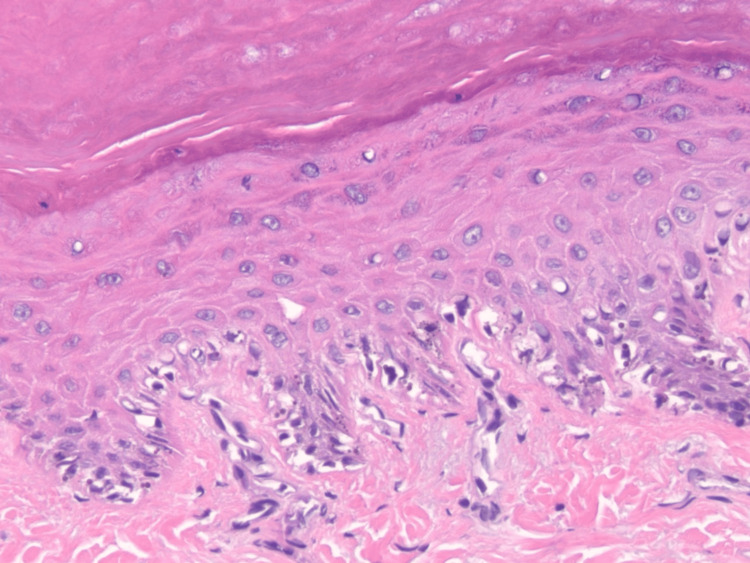
Hematoxylin and eosin (H&E) stain, ×10 magnification. Acral epidermis displays mild acanthosis with elongated rete ridges. Basal keratinocytes exhibit abundant coarse brown pigment granules; no melanocytic confluence, pagetoid spread, or cytologic atypia is identified.

**Figure 3:**
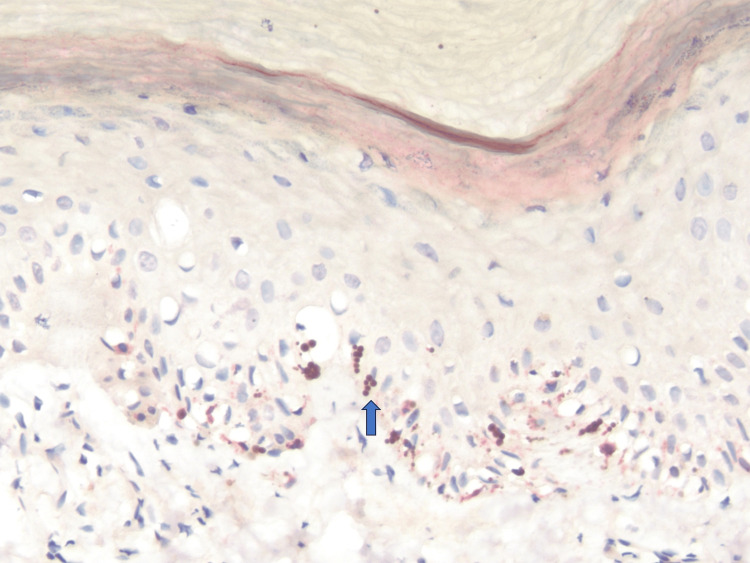
Immunohistochemistry for Melan-A and SOX10, ×10 magnification. Sparse, evenly distributed melanocytes are highlighted at the dermoepidermal junction, confirming physiologic density and distribution without clustering or upward migration.

Histopathologic findings of hematoxylin-eosin sections demonstrated acral epidermis with compact orthokeratosis and preserved ridge‑furrow architecture (Figure 2). The basal layer contained increased melanin pigment without evidence of confluent junctional melanocytic proliferation. Numerous coarse, oversized, dark brown granules were present within basal and immediate suprabasal keratinocytes, consistent with macromelanosomes. Melanocytes, highlighted by Melan‑A and SOX10, were present at normal density along the basal layer, without nesting, pagetoid spread, or cytologic atypia (Figure 3). There was no associated lichenoid/interface dermatitis, elastosis, or significant dermal melanophage accumulation. The superficial dermis contained only sparse perivascular lymphocytes and no fibrosis.

**Figure 4:**
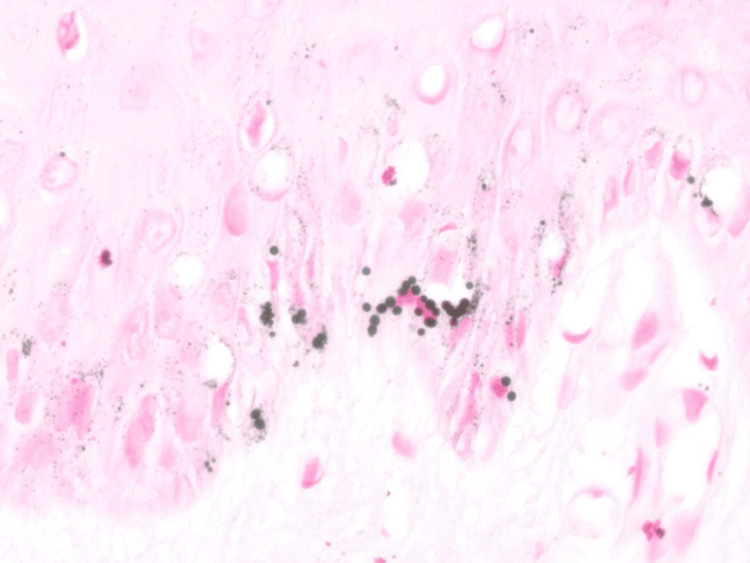
Fontana–Masson stain, ×20 magnification. Basal keratinocytes contain coarse, irregularly shaped, black-staining granules consistent with macromelanosomes. Pigment is confined to the epidermis without dermal melanophages or pigment incontinence.

Special staining with Fontana‑Masson highlighted abundant coarse melanin granules within keratinocytes, while Perls’ Prussian blue stain was negative for iron deposition, thereby excluding hemosiderin‑type pigment and minocycline‑related mixed pigmentation (Figure 4). PRAME (preferentially expressed antigen in melanoma) immunohistochemistry was negative.

The low melanocyte density at the dermoepidermal junction, coupled with the absence of epidermal effacement and proinflammatory changes, argues against acral melanoma in situ or regression of an atypical melanocytic proliferation. The dominant microscopic feature is keratinocyte macromelanosomes within an otherwise unremarkable acral epidermis.

## Discussion

Pigmented lesions of acral skin require judicious evaluation because of the diagnostic difficulty in distinguishing benign lesions from early melanoma. Our patient presented with a new pigmented macule on the plantar foot at age 77, raising concern for ALM. However, histologic evaluation revealed a benign process characterized by hyperpigmentation of the basal cell layer with a normal number of melanocytes and numerous macromelanosomes.

Comprehensive reviews of acral melanocytic lesions and macromelanosomes provide a broader context for these findings [4-6] while also highlighting the spectrum of PRAME expression in acral melanocytic lesions [7,8] and the molecular mechanisms underlying melanin transfer and formation of macromelanosomes [9].

Differential diagnosis

The clinical differential for acral pigmentation is extensive; only entities directly relevant to our histologic pattern are touched on here.

Acral lentiginous melanoma in situ (ALM‑IS) [4] is characterized by increased melanocyte density along the junction (often one melanocyte per basal keratinocyte or more), confluence of melanocytes, pagetoid scatter, and lentiginous cytologic atypia. PRAME expression is typically diffuse and strong in melanoma [8]. Our lesion lacked these features: melanocyte density was normal, distribution was even, and PRAME was negative. Furthermore, the coarse pigment was confined to keratinocytes rather than melanocytes, which is not typical of melanoma.

Acral lentigo or lentigo simplex [4,5] clinically presents as flat brown macules, not unlike our case presentation. However, histologically, they show elongated rete ridges with basal hyperpigmentation and mild melanocyte proliferation, which is not seen in our case. Macromelanosomes are not typically a feature. Our lesion lacked rete elongation beyond that expected in acral skin and displayed macromelanosomes.

Frictional dermatoses/friction melanosis [6,7] is clinically linked to repetitive mechanical stress, frequently on extensor or contact surfaces; histology may show pigment incontinence and compact ortho‑parakeratosis with variable granular layer changes. Our case lacks a convincing friction history and shows macromelanosomes without dermal melanophages.

Post‑inflammatory hyperpigmentation (PIH) [9] often follows inflammation, dermatitis, trauma, or other insults. Histologically, PIH shows increased melanin in basal keratinocytes with prominent pigment incontinence: melanophages and hemosiderin-laden macrophages in the upper dermis. None of these were present in our lesion. The patient had no history of preceding inflammation.

Solar lentigo or actinic melanosis [5] should be considered in skin lesions of older patients, exhibiting elongation of rete ridges, basal hyperpigmentation, mild melanocyte proliferation, and solar elastosis. Macromelanosomes may occur at the periphery of lentigo maligna or in actinic damage. Our lesion lacked solar elastosis and was on a UV‑protected site.

Congenital melanocytic nevus is excluded due to the absence of melanocytic nests or dermal melanocytes.

Genetic pigmentary disorders (e.g., Chediak-Higashi, Griscelli syndrome) [10,11] are characterized by defects in melanosome formation or transport. Macromelanosomes are present throughout the skin and hair. Patients often have systemic manifestations such as immunodeficiency, neurologic abnormalities, or ocular findings. Our patient lacked systemic features, and the lesion was acquired and solitary.

Drug‑induced hyperpigmentation (minocycline, hydroxyurea, and antimalarials) usually presents with slate‑gray or blue‑gray discoloration; histology may reveal both melanin and iron deposition or dermal macrophage pigment. The focal nature of changes, negative iron stain, and absence of clinical history of the above medications argue against such interpretation.

We employ the term "acral melanosis with macromelanosomes" as a descriptive, provisional label to flag a benign pattern when the triad is present. Melanocytic macule, Laugier-Hunziker syndrome, and Peutz-Jeghers syndrome are typically site-specific or syndromic and therefore clinically distinct from the current presentation. Café-au-lait macule (CALM) is a theoretical consideration; however, CALM is unlikely given the absence of syndromic features, the adult-onset acral site, the mottled clinical pigmentation, and a histologic pattern dominated by keratinocyte macromelanosomes with normal melanocyte density and architecture (Melan-A/SOX10), rather than the uniform basal hypermelanosis typical of CALM.

Pathogenesis of macromelanosomes

Macromelanosomes may form when melanosomes fail to divide properly or when melanin synthesis continues without normal segregation into smaller granules [11]. In congenital disorders, mutations in genes regulating melanosome transport (e.g., LYST in Chediak-Higashi, RAB27A in Griscelli) result in giant melanosomes. In acquired lesions, the mechanism is less well understood. Some authors propose that mechanical stress and friction on acral skin may disrupt the transfer of melanosomes from melanocytes to keratinocytes. The patient’s lesion was located on the plantar foot, a weight‑bearing site subject to chronic pressure and shear forces, which might impair melanosome transfer and lead to the accumulation of oversized granules.

Alternatively, chemical exposure from adhesives, footwear materials, or topical medications could interfere with melanosome packaging and distribution. The patient denied known exposures, but subtle environmental factors cannot be excluded. Age-related alterations in keratinocyte melanocyte signaling may also contribute. Keratinocytes regulate melanocyte growth and melanin transfer via paracrine factors such as endothelin‑1, stem cell factor, and α-melanocyte-stimulating hormone. With aging, changes in these signals could lead to abnormal melanosome formation or retention.

The lack of melanocyte proliferation suggests that the stimulus did not trigger melanocytic hyperplasia but rather affected melanin synthesis or transfer. Macromelanosomes located in keratinocytes and melanocytes support the idea that keratinocyte metabolic defects may play a role.

Proposed nomenclature and AI provenance

We propose the term “Acral Melanosis with Macromelanosomes (AMM)” for a benign acral pigmentary alteration defined by conspicuous keratinocyte macromelanosomes with otherwise normal melanocyte density and architecture. The phrase and initial construct were generated collaboratively with ChatGPT 5 (an AI large language model) during iterative expert-guided prompting and editing. To our knowledge, based on a targeted PubMed review of AI in pathology literature and pigmentary disorders performed on August 23, 2025, this represents the first pathological entity to be named and provisionally defined with direct AI input and then documented as a case report. We encourage explicit “AI provenance statements” in manuscripts when AI contributes to terminology or hypothesis generation.

Provisional diagnostic criteria

Per our case presentation and the use of ChatGPT-5, the provisional diagnostic criteria for AMM can be seen in Table 1.

**Table 1:** Diagnostic criteria for acral melanosis with macromelanosomes

Criteria type
Major (all required):
1. Acral (palmar/plantar or subungual) macule/patch with a stable or indolent course
2. Histology showing conspicuous keratinocyte macromelanosomes in basal/suprabasal layers
3. Normal basal melanocyte density without junctional nesting, pagetoid spread, or cytologic atypia on Melan‑A/SOX10
4. Fontana‑Masson positive; Perls’ iron negative
5. Absence of postinflammatory/regressive changes
Minor (supportive):
1. Lesion arising on plantar or palmar weight‑bearing acral skin
2. Occurring in elderly patients, where chronic pressure and age‑related melanocyte dysfunction may impair melanosome transfer
3. Dermoscopy lacks a parallel ridge pattern

Clinical significance

The main clinical importance of this entity lies in its potential to mimic early melanoma and thus lead to unnecessary wide excision. Awareness of AMM allows the pathologist to confidently diagnose a benign condition when histologic criteria and immunohistochemistry support such an interpretation. It also underscores the importance of correlating histologic findings with clinical and dermoscopic features.

Because the lesion was completely removed with the biopsy, no further treatment was required. The patient was reassured and advised to monitor for any new lesions. Periodic skin examinations were recommended, especially given his age and the location of the lesion on the foot.

## Conclusions

We report a single benign acral pigmentary lesion characterized by conspicuous keratinocyte macromelanosomes, normal basal melanocyte density and architecture (Melan-A/SOX10), Fontana-Masson positivity, Perls negativity, and no histologic features of melanoma. We use the descriptive, provisional term acral melanosis with macromelanosomes for this pattern, which may clinically or histologically mimic early ALM. Key diagnostic features include normal melanocyte density, absence of atypia or pagetoid spread, presence of coarse melanin granules (macromelanosomes), and absence of post-inflammatory or regressive changes. Recognition of this pattern may help prevent overdiagnosis and unnecessary aggressive management. Larger case series will be needed to clarify its pathogenesis and the potential contribution of mechanical or chemical factors.
